# NK cell function is markedly impaired in patients with chronic lymphocytic leukaemia but is preserved in patients with small lymphocytic lymphoma

**DOI:** 10.18632/oncotarget.12097

**Published:** 2016-09-17

**Authors:** Helen M. Parry, Tom Stevens, Ceri Oldreive, Bassier Zadran, Tina McSkeane, Zbigniew Rudzki, Shankara Paneesha, Caroline Chadwick, Tatjana Stankovic, Guy Pratt, Jianmin Zuo, Paul Moss

**Affiliations:** ^1^ Institute of Immunology and Immunotherapy, College of Medical and Dental Sciences, University of Birmingham, B15 2TT, UK; ^2^ Institute of Cancer and Genomic Sciences, College of Medical and Dental Science, University of Birmingham, B15 2TT, UK; ^3^ Department of Haematology, Birmingham Heartlands Hospital, Birmingham, B9 5SS, UK; ^4^ Biomedical Services Unit, University of Birmingham, B15 2TT, UK

**Keywords:** NK cells, chronic lymphocytic leukaemia, small lymphocytic lymphoma, immunomodulation

## Abstract

Chronic lymphocytic leukemia (B-CLL) and small lymphocytic lymphoma (SLL) are part of the same disease classification but are defined by differential distribution of tumor cells. B-CLL is characterized by significant immune suppression and dysregulation but this is not typical of patients with SLL. Natural killer cells (NK) are important mediators of immune function but have been poorly studied in patients with B-CLL/SLL. Here we report for the first time the NK cell phenotype and function in patients with B-CLL and SLL alongside their transcriptional profile. We show for the first time impaired B-CLL NK cell function in a xenograft model with reduced activating receptor expression including NKG2D, DNAM-1 and NCRs in-vitro. Importantly, we show these functional differences are associated with transcriptional downregulation of cytotoxic pathway genes, including activating receptors, adhesion molecules, cytotoxic molecules and intracellular signalling molecules, which remain intact in patients with SLL. In conclusion, NK cell function is markedly influenced by the anatomical site of the tumor in patients with B-CLL/SLL and lymphocytosis leads to marked impairment of NK cell activity. These observations have implications for treatment protocols which seek to preserve immune function by limiting the exposure of NK cells to tumor cells within the peripheral circulation.

## INTRODUCTION

Chronic lymphocytic leukemia (B-CLL) and small lymphocytic lymphoma (SLL) have identical morphological and immunophenotypic features on tissue biopsy and are grouped together within the World Health Organization classification of hematological malignancy (WHO 2008) [1]. B-CLL is defined by peripheral blood lymphocytosis, bone marrow infiltration, and a variable degree of lymph node expansion [2, 3]. In contrast, patients with SLL do not demonstrate lymphocytosis and have a low incidence of cytopenias secondary to bone marrow infiltration [2]. The mechanisms underlying this differential distribution of tumor cells are not known but are likely to relate to fundamental properties of the tumor cell, such as chemokine receptor expression [4]. SLL is approximately ten times less common than B-CLL and accounts for 6% of all Non-Hodgkin lymphoma [5].

B-CLL is associated with a range of immunological complications including an increased propensity to infection and auto-immune complications [3, 6, 7]. In contrast, these complications are not a major feature of SLL [8] and most likely reflect differences in the tissue distribution of the tumor cells rather than the overall tumor load. A number of immunological abnormalities have been described in patients with B-CLL including hypogammaglobulinaemia and T cell dysfunction [9]. Natural killer cells (NK cells) are innate lymphocytes that play a critical role in the immune surveillance against infection and malignancy and also contribute to the therapeutic activity of current treatment protocols for B-CLL/SLL [10]. However, the phenotype and function of NK cells in patients with B-CLL and SLL have been relatively poorly investigated. Tumors evolve many mechanisms to evade elimination by NK cells [11, 12], and defects in the cytotoxic capacity of NK cells from patients with B-CLL were described as early as 1996 [13]. The mechanisms that underlie this phenotype remain uncertain but are thought to reflect an alteration in the balance between activatory and inhibitory signaling [14–16].

We investigated the *in vitro* and *in vivo* function of NK cells from patients with B-CLL and SLL and observed a selective and marked functional impairment in cells taken from patients with B-CLL. Global downregulation of several activating receptors, including NKG2D, DNAM-1 and NCRs, was observed on NK cells from patients with B-CLL. Using whole genome transcription microarray of NK cells, the transcription of many genes involved in cytotoxic function was also found to be dysregulated. These data reveal a profound and selective impairment of NK cell function in patients with B-CLL compared to those with SLL. The differential distribution of the B-CLL/SLL tumor within blood is therefore a critical determinant of NK cell function. These data are relevant to the potential detrimental influence of lymphocytosis during ‘watch and wait’ clinical monitoring or during treatments with targeted therapies that mobilize tumors cells into the bloodstream.

## RESULTS

### NK cells from patients with B-CLL demonstrate functional impairment during assays of *in vitro* and *in vivo* activity

**Figure 1:**
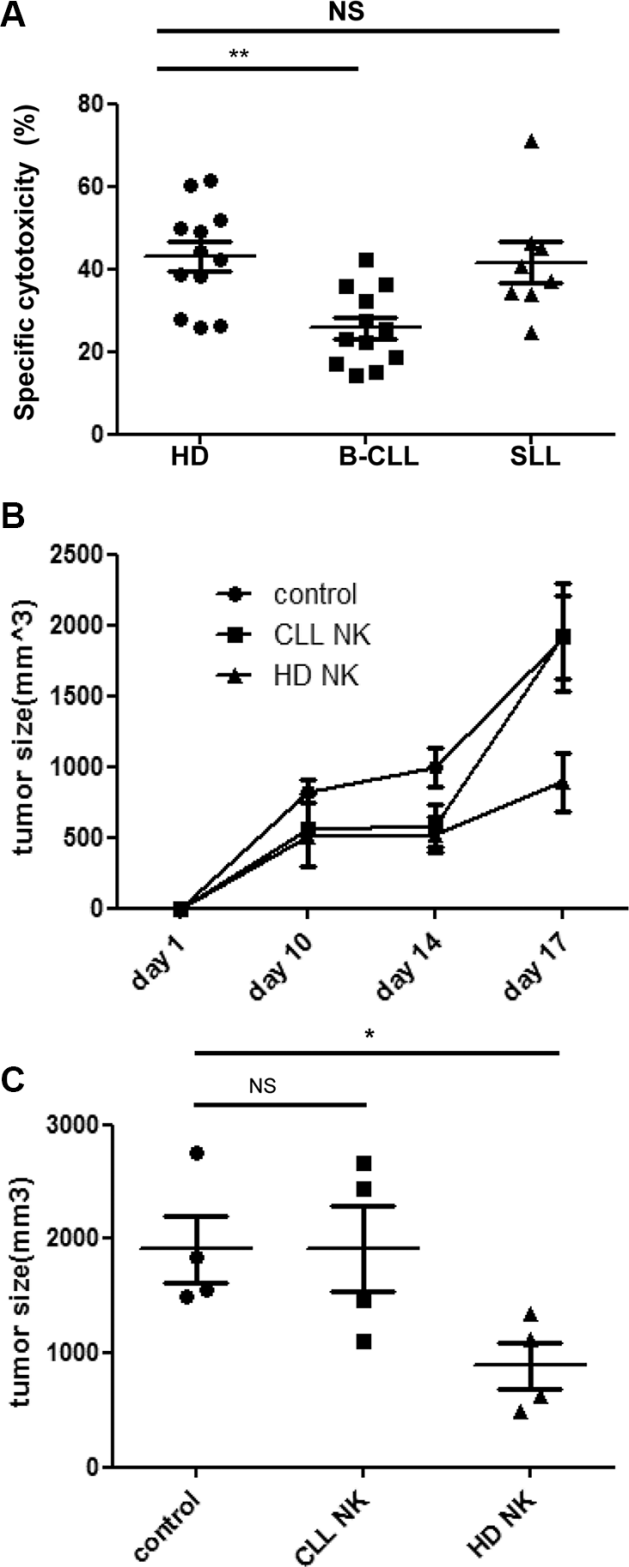
NK cells from patients with B-CLL fail to control tumor growth *in vitro* and *in vivo* (**A**): NK cells were enriched and co-cultured with CFSE-labelled K562 cells for 16hrs. The cytotoxicity was calculated according to the relative cell count of the live populations of target cells using flow cytometry. Data shown are mean values of the specific cytotoxicity from 12 healthy donors (HD), 12 patients with B-CLL and 8 patients with SLL. Error bars represent standard errors and significance was determined using Mann-Whitney testing. *p* < 0.01(**). (**B–C**): NOG (NOD/Shi-scid/IL-2Rg) mice were injected subcutaneously with 1 × 107 K562 cells and divided into 3 groups which were given either IL-2 only (control), IL-2 with HD-NK cells or IL-2 with CLL-NK cells. Tumour growth was monitored and measured regularly. The tumour growth curve (B) of the three groups are shown as mean values of the tumour size from 4 mice at different time points in each group, error bars represent standard errors. The tumour size at day 17 was compared among three groups (C). Error bars represent standard errors and significance was determined using Mann-Whitney testing. *p* < 0.05(*).

In order to investigate the functional capacity of NK cells taken from patients with B-CLL, an *in-vitro* cytotoxicity assay was carried out using the NK cell target line K562 [17]. NK cells were isolated from healthy donors (HD-NK) or patients with B-CLL (CLL-NK) prior to incubation with CFSE-labeled K562 cells. 43% of target cells were lysed following incubation with HD-NK cells (mean ± SEM: 43% ± 3.5%) but this was reduced by 40% following incubation with CLL-NK (mean ± SEM: 25.8% ± 2.6; *p* = 0.0017) (Figure 1A). This result has been confirmed by using Europium release based cytotoxicity assay (Supplementary Figure S1). In contrast, NK cells from patients with SLL demonstrated no significant difference in their lytic capacity compared to NK cells from HD (mean ± SEM: 41.7% ± 4.9; *P* = 0.56) (Figure 1A).

In order to assess how this impairment *in vitro* function was translated into activity *in vivo* we next used a xenograft model of NK cytotoxicity. NOG mice were injected subcutaneously with K562 cells and then at day 3 NK cells, from either HD or patients with B-CLL, were infused. IL-2 was given to support NK cell expansion and a control group of mice received IL-2 treatment alone. K562 tumor growth became apparent in all mice at day 7 after injection and tumor size was measured on day 10, 14 and 17 (Figure 1B). NK cells taken from HD substantially reduced the growth of the K562 tumor such that tumor volume was suppressed by 54% at day 17. Tumor sizes derived from control mice were 1910 ± 290 mm3 (mean ± SEM) compared to 890 ± 200 mm3 in those mice infused with HD-NK cells (*p* = 0.029) (Figure 1C). In contrast, NK cells taken from patients with B-CLL were incapable of any significant degree of tumor suppression (Figure 1C).

### NKG2D expression and NKG2D-mediated cytotoxic function are both decreased in NK cells taken from patients with B-CLL but not SLL

**Figure 2:**
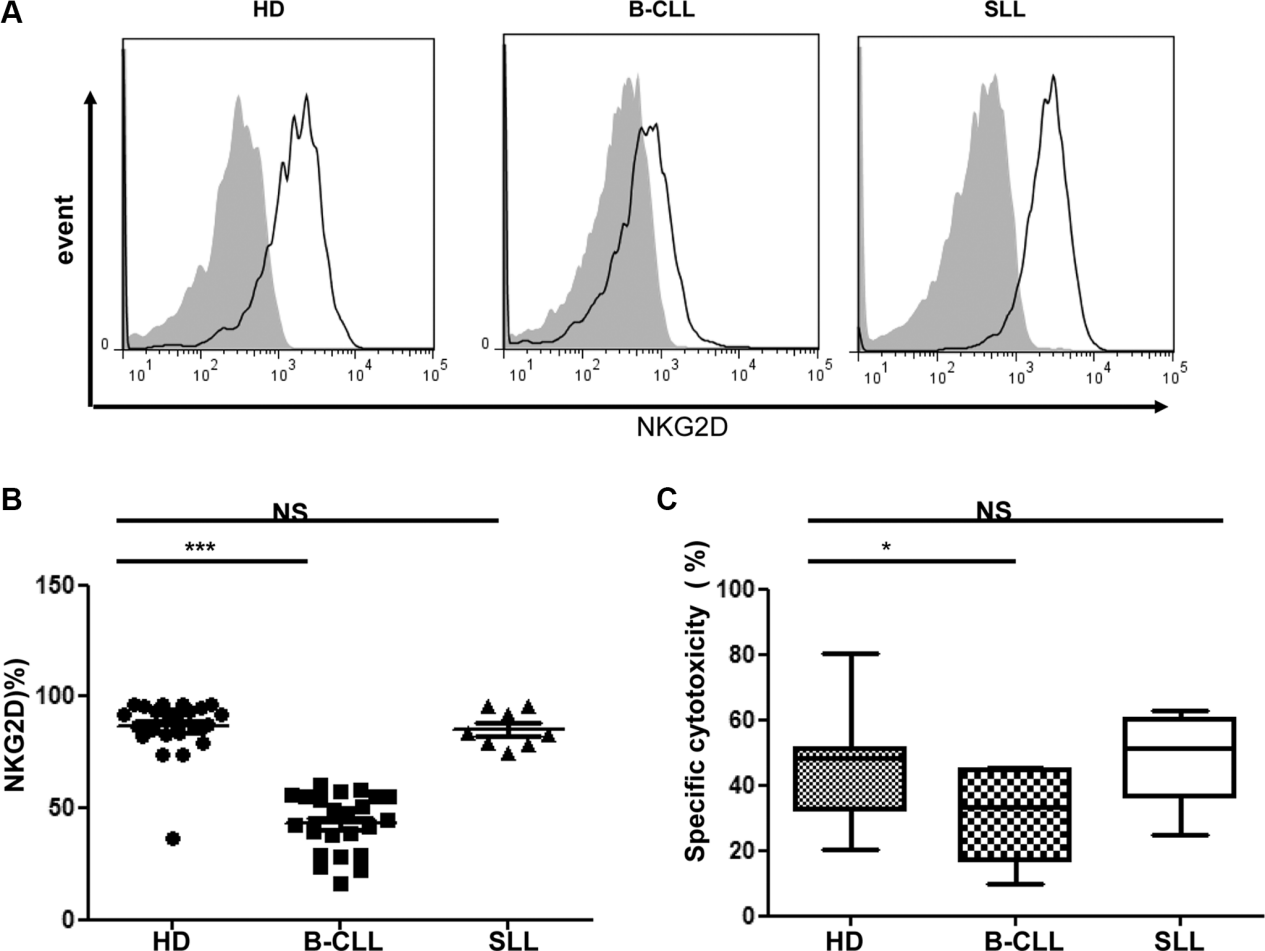
Expression of NKG2D on NK cells is downregulated in patients with B-CLL but not patients with SLL (**A)** An example of flow cytometry staining of the surface NKG2D expression on NK cells from one healthy donor (HD), one patient with B-CLL and one patient with SLL. Solid black lines represent NKG2D staining and grey-filled histograms represent isotype control staining. (**B**) The NKG2D surface expression on NK populations from 23 HD, 23 patients with B-CLL and 8 patients with SLL were studied. The percentage of NKG2D positive NK cells is shown, with error bars representing standard errors and the significance determined using Mann-Whitney testing, *p* < 0.001(***). (**C**) For the NKG2D specific NK cytotoxicity assay, a mixture of NKG2D ligand-transfected CHO cells and control transfected CHO cells were co-incubated with effectors for 16 hrs. After co-culture, the ratio of control CHO cells to NKG2DL-transfected CHO cells was calculated to determine the percentage of specific killing. Data shown are median values of specific cytotoxicity from 7 HD, 7 patients with B-CLL and 6 patients with SLL. Bars represent minimal and maximal values of the data and significance was determined using Mann-Whitney testing, *p* < 0.05(*).

NK cell cytotoxicity is mediated through a range of activating receptors, of which NKG2D-mediated signaling is a dominant pathway. As such, we next went on to determine the surface expression of NKG2D on NK cells taken from HD and patients with B-CLL (*n* = 23). A markedly reduced expression of NKG2D was observed on NK cells from patients with B-CLL but not SLL, in comparison to the profile on cells from HD (Figure 2A). In particular, the percentage of NKG2D-positive NK cells was reduced by 51% amongst patients with B-CLL (mean ± SEM B-CLL 43.1% ± 2.7% vs HD 86.6% ± 2.7%; *p* < 0.001; Figure 2B). Interestingly, the percentage of NKG2D positive NK cells was not reduced in patients with SLL (mean ± SEM 85.3% ± 2.9%) in comparison to that observed on NK cells from HD (Figure 2B).

In order to assess if this reduction of NKG2D surface expression on NK cells from patients with B-CLL is functionally significant we then determined the ability of NK cells to mediate NKG2D-dependent cytotoxicity. Specifically, a CHO cell line was stably transfected with ULBP6, an NKG2D ligand, and was used as a target cell, whilst untransfected CHO cells served as a negative control. The cytotoxicity to this cell line has been shown to be able to be blocked by anti-NKG2D antibody (Supplementary Figure S4). NKG2D-mediated killing was substantially reduced using NK cells from patients with B-CLL in comparison to either NK cells taken from HD or patients with SLL. In particular, specific lysis of the ULBP6-expressing CHO cell line by NK cells from HD was 47% compared to only 29% using NK cells taken from patients with B-CLL (mean ± SEM: HD 47% ± 7.0% vs B-CLL, 29% ± 5.4% *p* < 0.05) (Figure 2C). Again, NK cells taken from patients with SLL did not reveal any impairment of lytic activity compared to HD (mean ± SEM SLL 48.5% ± 6.1%) (Figure 2C).

### The expression of DNAM-1 and natural cytotoxicity receptors are also reduced on NK cells from patients with B-CLL but not SLL

**Figure 3:**
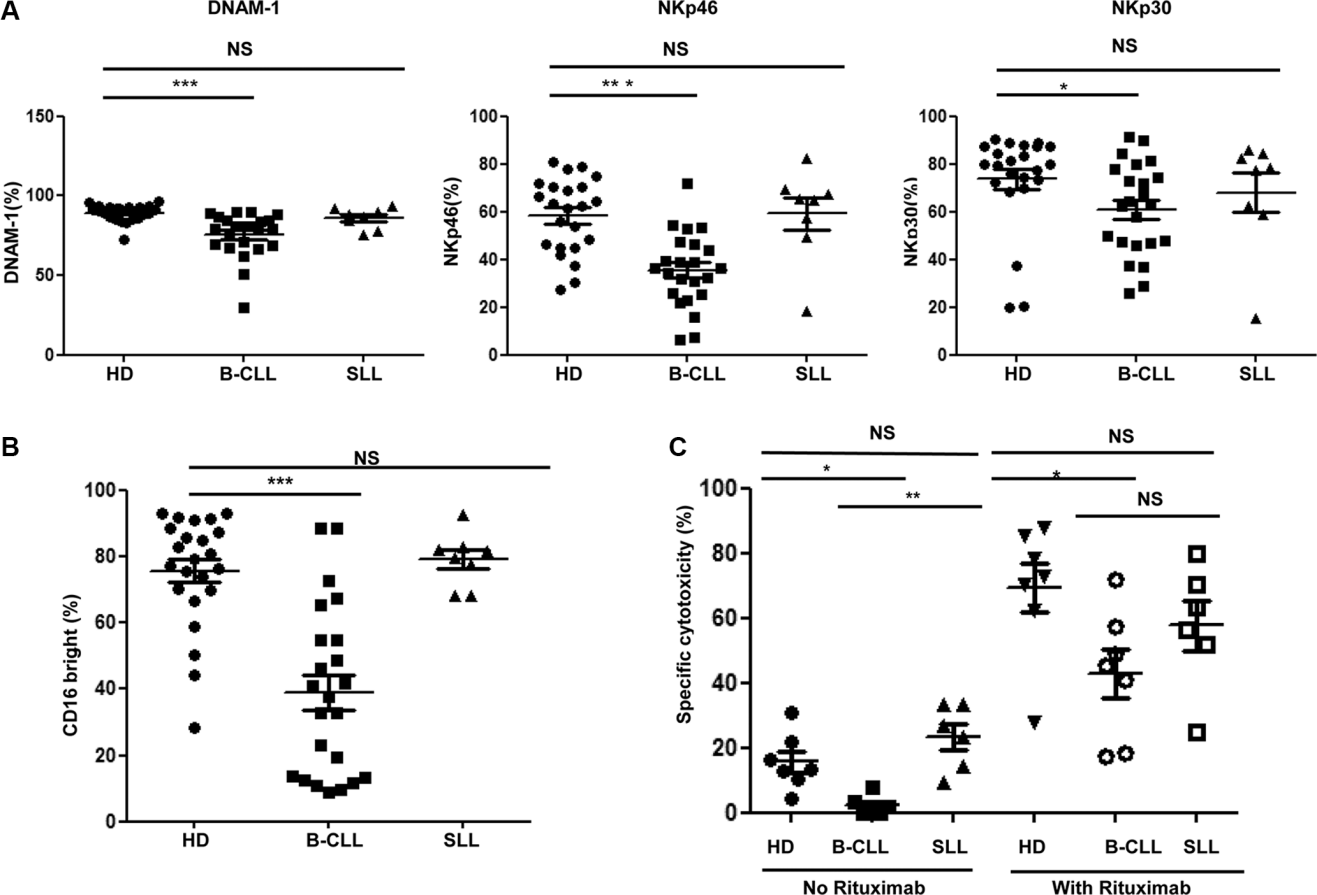
Expression of DNAM-1, natural cytotoxicity receptors and CD16 are also reduced on NK cells from patients with B-CLL (**A**) The surface expression of DNAM-1 (left panel), NKp46 (middle panel) and NKp30 (right panel) was determined on NK cells from 23 healthy donors (HD), 23 patients with B-CLL and 8 patients with SLL were studied using flow cytometry. Data shown are mean values for the percentage of cells positive for each receptor within HD, patients with B-CLL and patients with SLL. Error bars represent standard errors and significance was determined using Mann-Whitney testing, *p* < 0.05(*) and *p* < 0.001(***). (**B**) The expression of CD16 was determined on NK cells from 23 HD, 23 patients with B-CLL and 8 patients with SLL using flow cytometry. Data shown are mean values of the percentage of CD16 bright populations of NK cells from HD, patients with B-CLL and patients with SLL. Error bars represent standard errors and significance was determined using Mann-Whitney testing, *p* < 0.001(***). (**C**) For the rituximab induced NK cytotoxicity assay, B-CLL primary tumor cells were enriched and used as target cells. The target cells were then incubated NK cells, with or without Rituximab, for 16 hours. Specific lysis was calculated according to the relative cell counting using flow cytometry. Data shown are mean values of the specific cytotoxicity by NK cells from 7 HD, 7 patients with B-CLL and 6 patients with SLL. Error bars represent standard errors and significance was determined using Mann-Whitney testing, *P* < 0.05(*), and *P* < 0.01(**).

In addition to NKG2D, several other activating receptors are expressed on NK cells, including DNAM- 1 and the natural cytotoxicity receptors (NCR) NKp30 and NKp46. The surface expression of DNAM-1, NKp30 and NKp46 was examined on NK cells taken from HD and patients with B-CLL or SLL. All three activating receptors were markedly downregulated on NK cells from patients with B-CLL compared to NK cells from HD. Of note, the percentage of DNAM-1 positive NK cells was 89.3% ± 1.1% on HD-NK compared to 75.5% ± 2.9% on CLL- NK cells (*p* < 0.001) (Figure 3A left panel). Similarly, the percentage of NKp46 positive NK cells was 58.3% ± 3.3% on HD-NK compared to 35.4% ± 3.2% on CLL-NK cells (*p* < 0.001) (Figure 3A middle panel). Also the percentage of NKp30 positive NK cells was 73.6% ± 4.2% and 61.1% ± 4.0% (*p* = 0.0152) on HD-NK and CLL-NK cells respectively (Figure 3A right panel). In contrast, the percentage of these receptors positive NK cells from patients with SLL was not significantly different from those found on HD-NK (DNAM-1, 86.0% ± 2.3%; NKp46, 59.2% ± 6.7%; NKp30, 68.0% ± 8.3%) (Figure 3A). Together, these data show that expression of activating receptors is substantially reduced on NK cells taken from patients with B-CLL, but not from patients with SLL. Also the mean fluorescence intensity (MFI) of surface expression of NKG2D, DNAM-1 and NCRs showed similar trend of reduction on NK cells from patients with B-CLL, but not from patients with SLL (Supplementary Figure S3).

### NK cells from patients with B-CLL express lower levels of CD16 and display impaired antibody-dependent cell mediated cytotoxicity (ADCC)

The Fc receptor, CD16, is an activating receptor expressed on the majority of NK cells and plays an important role in mediating ADCC. The pattern of CD16 expression was next examined on NK cells from patients with B-CLL and SLL (gating strategy shown in Supplementary Figure S5). 75.7% ± 3.5% of NK cells from HD were CD16^bright^, compared to only 39.0% ± 5.3% of NK cells derived from patients with B-CLL (*p* < 0.001) (Figure 3B). The percentage of CD16^bright^ NK cells in patients with SLL (79.1% ± 2.9) was not different to HD-NK cells (Figure 3B).

ADCC is an important effector mechanism underlying the activity of rituximab, a CD20-specific monoclonal antibody used in the management of B-CLL [18]. In order to assess if the reduction in the proportion of CD16^bright^ NK cells from patients with B-CLL might impact on rituximab efficacy, a rituximab-sensitized ADCC *in vitro* assay was undertaken, with primary allogeneic B-CLL tumor cells used as targets. In the absence of rituximab, killing of B-CLL tumor cells was relatively modest regardless of the source of NK cells, reflecting natural resistance of primary B-CLL tumor cells to NK cell mediated lysis. However, even here tumor cell killing was reduced using NK cells from patients with B-CLL (2.2% ± 1.3) compared to NK cells from HD (15.7% ± 3.2) or patients with SLL (23.4% ± 4.0) (Figure 3C). The addition of rituximab led to a substantial increase in tumor lysis by all three NK effector subgroups (they are all allogeneic NK cells against a single clone of primary tumor cells). However, although tumor killing by CLL-NK cells increased by nearly 20- fold following rituximab, from 2.2% to 43%, this was still significantly lower than the lysis observed with rituximab in combination with NK cells from HD and patients with SLL (69% and 58% respectively) (Figure 3C). Similar results has been obtained using the enriched B cells from healthy donors as target cells (Supplementary Figure S6). These results indicate that allogeneic NK cells from patients with B-CLL demonstrate reduced lysis of primary B-CLL cells, which is partially but not completely, enhanced in the setting of ADCC.

### The transcriptional profiles of NK cells from patients with either B-CLL or SLL display a unique profile

To investigate the transcriptional basis for the functional and phenotypic impairment of NK cells from patients with B-CLL, we used GeneChip^®^ Human Transcriptome Array 2.0. and performed comparative transcriptional profiling of NK cells taken from HD or patients with either B-CLL or SLL (*n* = 5 in each group). 70523 positive probe-sets were found among the whole chip-set.

**Figure 4:**
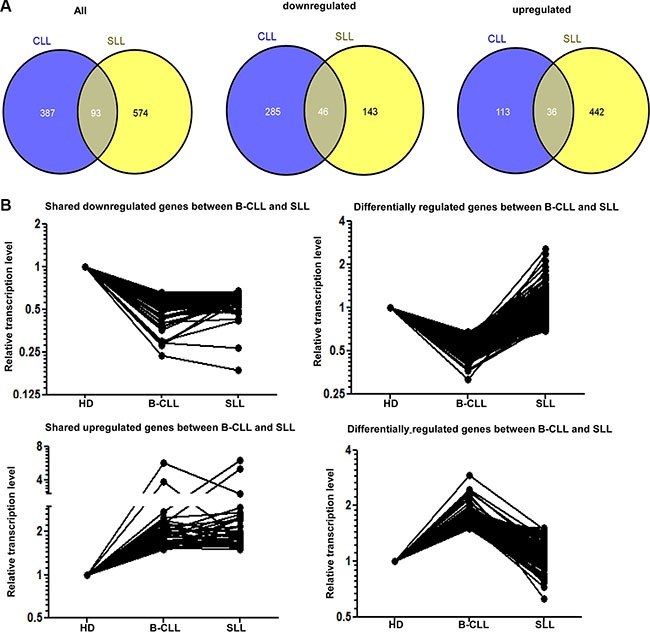
The transcription profile of NK cells in patients with B-CLL or SLL compared to healthy donors (**A**) Venn diagram demonstrating the intersection of genes significantly modified in NK cells from patients with B-CLL or SLL compared to healthy donors (HD) in microarray analysis. The number of genes which show any significant change is shown in the left panel and the number of downregulated or upregulated genes are shown in the middle and right panels respectively. (**B**) Graphical representation of the relative transcriptional level of similarly or differentially expressed genes in NK cells from patients with B-CLL or SLL compared to HD. 46 genes are downregulated in NK cells from both patients with B-CLL and SLL compared with HD (top left graph) and 36 genes are upregulated in these groups (bottom left graph). 285 genes are downregulated in NK cells from patients with B-CLL compared to HD but not in patients with SLL (top right graph) whereas 113 genes are upregulated in NK cells from patients with B-CLL compared to HD, but not in patients with SLL (bottom right graph).

The pattern of gene expression within NK cells from patients with B-CLL or SLL was compared to the transcriptional profile observed in NK cells taken from HD. 480 and 667 genes showed at least a 1.5 fold change in transcript levels within CLL-NK and SLL-NK samples respectively compared to HD-NK (*p* < 0.05). However, it was noteworthy that the great majority of these differences were not shared between patients with SLL and B-CLL. In particular, 331 genes were downregulated in CLL-NK samples and 189 genes were downregulated within the SLL-NK group. However, only 46 of these genes shared a common pattern of downregulation within both groups (Figure 4A middle panel, Figure 4B top panels). A further 149 and 478 genes demonstrated increased expression within CLL-NK and SLL-NK samples respectively, whilst only 36 genes were upregulated in both patient groups (Figure 4A right panel, Figure 4B bottom panels). Together, 93 shared genes were significantly modified within both groups compared to HD-NK (Figure 4A left panel). 46 of these shared genes were downregulated, 36 were upregulated in both groups and 11 differed in their direction between the two groups. DAVID functional annotation tool was then used to identify gene sets of GO (Gene ontology terms) that were enriched. Consistent transcription changes were observed within each sample of the three participant groups. The 93 genes included genes encoding phosphoproteins (38 genes), regulation of transcription (15 genes), intracellular signaling cascade (11 genes) and regulation of apoptosis (9 genes) (Figure 4A left panel and Supplementary Figure S7).

### Multiple genes involved in NK cytotoxicity are expressed at lower level in NK cells from patients with B-CLL

**Figure 5:**
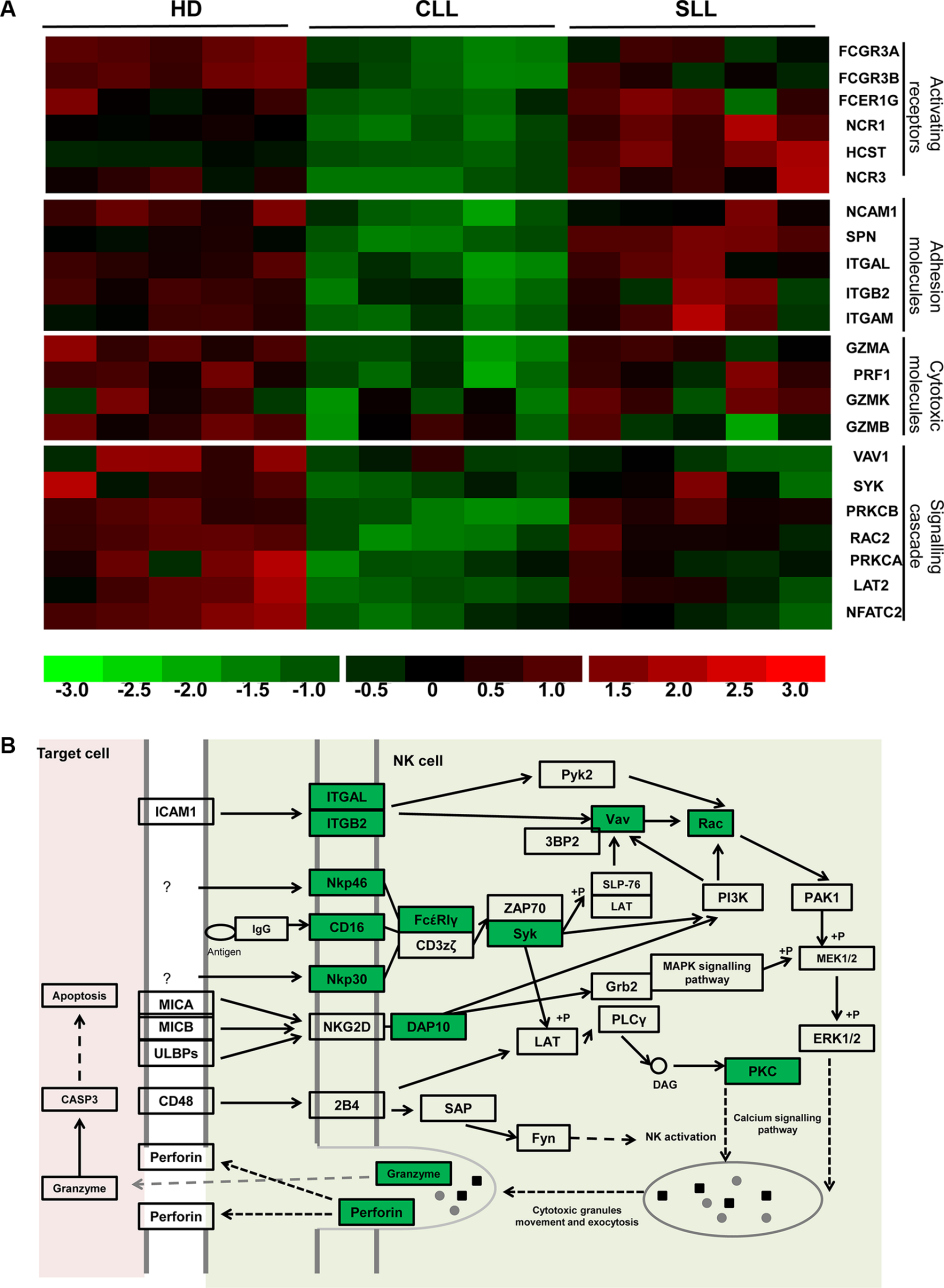
Multiple points in the cytotoxicity pathway of NK cells are impaired in patients with B-CLL (**A**) This demonstrates the relative intensity profile of 22 genes related to NK cytoxic function which are downregulated in NK cells from patients with B-CLL compared to HD. Each column represents one sample, whilst each row represents the relative hybridization intensity of an individual gene. The 22 transcripts were classified into 4 categories (From the top to the bottom): NK activating receptors (6 genes), adhesion molecules (5 genes), cytotoxic molecules (4 genes) and intracellular signal transduction related molecules (7 genes). The colour on the heatmap indicates the magnitude of the relative expression of genes across the samples, with brighter red indicating higher expression and brighter green indicating lower expression. (**B**) The NK cell cytotoxicity pathway (adapted from KEGG pathways analysis). Highlighted genes in green are those that have been shown to be significantly downregulated in patients with B-CLL compared to HD.

We next determined the biochemical pathways that are modulated by the pattern of trancriptional expression within NK cells. KEGG pathway analysis (http://www.genome.jp/kegg/pathway.html) highlighted the gene group responsible for NK cell-mediated cytotoxicity that were downregulated by more than 1.5 fold within CLL- NK samples compared to HD-NK (*p* < 0.05). These were then grouped into four classes, based on the NK cytotoxic pathway (Figure 5B), namely activating receptors (*NCR1, NCR3, FCGR3A, FCGR3B, FCER1G, HCST*), adhesion molecules (*NCAM1*, *ITGAL*, *ITGB2*, *SPN*, *ITGAM*), cytotoxicity molecules (*GZMA, GZMB, GZMK, PRF1*) and genes involved in the cytotoxic intracellular signaling cascade (*NFATC2, SYK, VAV1, RAC2, PRKCA, PRKCB, LAT2*) (Figure 5A). These data indicate that several biochemical pathways important for cytotoxic funcion are imparied in NK cells from patients with B-CLL.

The profile of these genes was next examined in NK cells taken from patients with SLL and, with the exception of *VAV1* which was reduced by 48% in patients with SLL, all genes were found to be expressed at the same level as cells from HD (Figure 5A). These data are consistent with the substantial difference in the pattern of functional cytotoxicity observed between NK cells taken from patients with B-CLL or SLL.

In order to assess how the transcriptional profile correlated with the pattern of surface expression, we next correlated microarray data with flow cytometric analysis. The relative transcription levels for *NCR1* (NKP46) and *NCR3* (NKp30) were 94 and 81 in HD-NK, 60 and 45 in CLL-NK and 123 and 93 in SLL-NK. As described above, these levels were significantly lower in CLL- NK cells compared to HD-NK and SLL-NK (*P* = 0.001 for NCR1 and 0.0002 for NCR3) with no difference observed between HD-NK and SLL-NK. Interestingly, flow cytometric analysis of surface expression of NKp46 and NKp30 revealed the same pattern, with MFI of 402 and 2650 respectively in HD, 337 and 2060 in patients with B-CLL and 432 and 2240 in patients with SLL (Supplementary Figure S3). A further 5 cytotoxic-related genes, (*NCAM1* (CD56), *FCGR3A* (CD16a), *FCGR3B* (CD16b), *PRF1* (perforin) and *GZMB* (granzyme B), also exhibited a strong correlation between transcriptional profile and surface or intracellular protein expression (data not shown).

Interestingly, the level of NKG2D transcription did not differ between CLL-NK and HD-NK cells. However, NKG2D is expressed as a dimer associated with the adaptor protein DAP10 [19, 20] and expression of the *HCST* gene, which encodes DAP10 was markedly reduced in CLL- NK cells compared with HD-NK and SLL-NK (mean transcription: HD-NK 240 vs CLL-NK 162 vs SLL- NK 428 respectively) (Figure 5A). The association with DAP10 is a pre-requisite for NKG2D expression at the cell surface [21] and DAP10 phosphorylation is required for the intracellular signaling that mediates NK cell activation [22, 23]. As such, the reduction in DAP10 transcription in CLL-NK cells is sufficient to account for the observation of decreased NKG2D expression and impaired NKG2D-specific killing by CLL-NK cells (Figure 2).

## DISCUSSION

NK cells have a critical role in the control of infection and malignant disease [24], but have been relatively poorly investigated in patients with B-CLL, despite the observation that both of these complications are increased in this disorder. Our study has revealed significant functional impairment of NK cells from patients with B-CLL and used for the first time transcriptional and flow cytometric analysis to reveal multiple mechanisms in NK cytotoxicity impairment. Additionally, we show that NK cell cytotoxicity was unimpaired in patients with SLL, a clinical disorder that is defined as part of the same disease entity as B-CLL.

Impaired cytolytic function of NK cells in patients with B-CLL has previously been described [13, 16, 25, 26]. This impairment has been attributed to a lack of azurophilic granules, impaired release of cytolytic molecules [13], and impaired expression of activating receptors [16]. In agreement with others, our study has confirmed the impaired cytotoxic function of NK cells from patients with B-CLL *in vitro* using K562 target cells but have further extended this observation *in vivo* using a xenograft model. Importantly, we also for the first time use an NKG2D-dependent cytotoxicity assay to demonstrate that reduced NKG2D expression directly correlated with impairment in NK cell cytotoxicity (Figure 2C).

Our microarray analysis contrasted the transcriptional profile of NK cells from HD or patients with either B-CLL or SLL and remarkably found that many genes, which regulate cytotoxicity are downregulated in NK cells taken from patients with B-CLL. These included NK activating receptors such as NKp30, NKp46 and several adhesion molecules. Adhesion molecules are required for immunological synapse formation, which has been reported to be impaired in patients with B-CLL and improves with the use of lenolidamide [27]. We also found significant reduction in expression of genes involved in cell signalling and cytotoxic granule formation. The combination of these defects would all contribute to the impaired cytotoxic function observed in NK cells from patients with B-CLL.

NK cells are believed to be important in the elimination of tumor cells. It is therefore not surprising that tumors evolve mechanisms to evade elimination by NK cells, including secretion of immunoregulatory molecules such as PGE2 and TGF-β [28, 29]. Confirming the findings of others, we incubated HD-NK cells with TGFβ-1 for 48hrs and found the surface expression of NKG2D was reduced by 53% (*p* = 0.014) (Supplementary Figure S8) [30, 31]. And also B-CLL tumor cells produce considerable amounts of TGF-β1, such that elevated levels are found in patient serum [32, 33]. Soluble TGF-β1 therefore represents one mechanism by which the functional impairment of NK cells in patients with B-CLL may arise.

**Figure 6:**
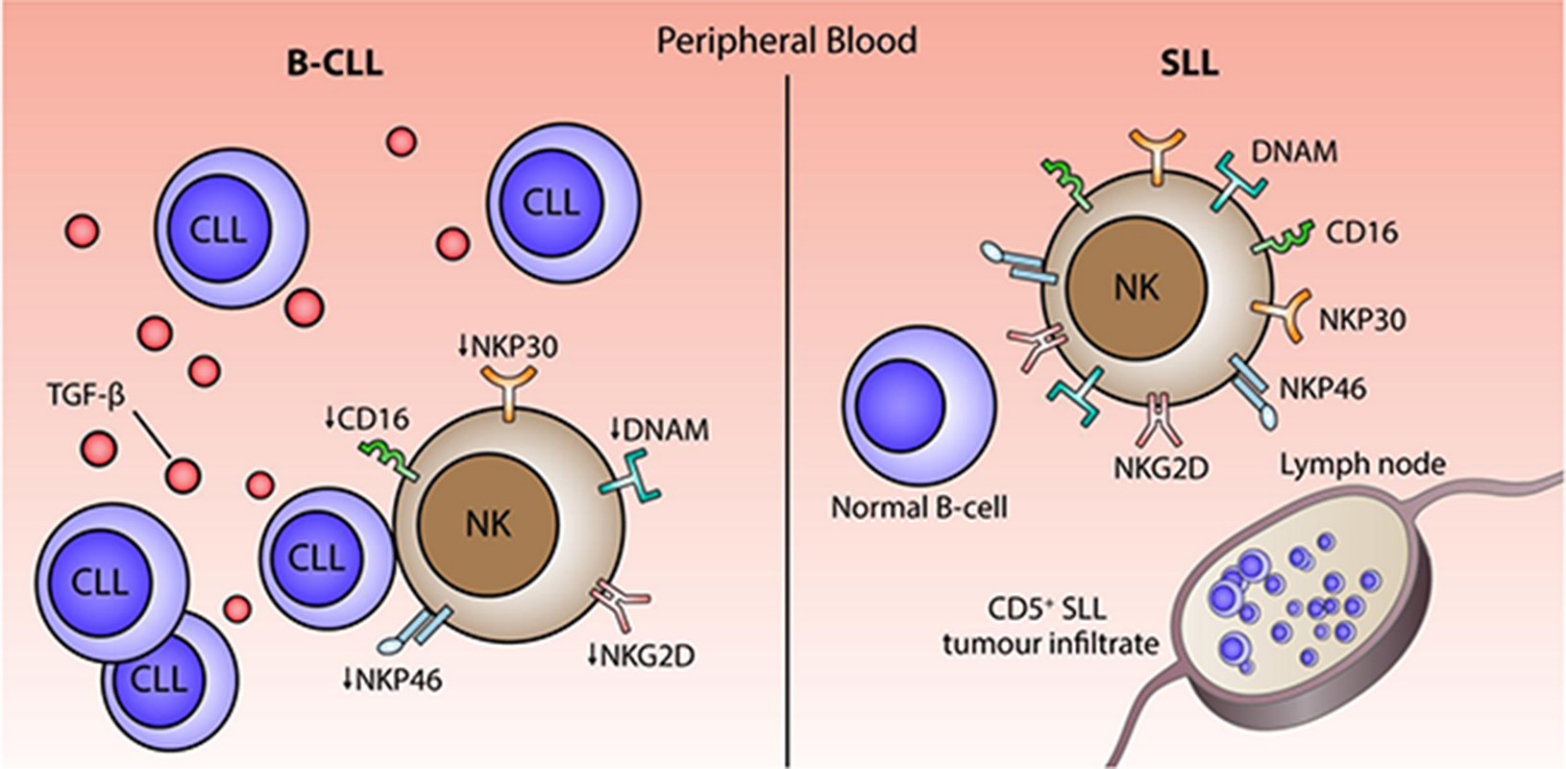
Model of the potential mechanisms that underlie impairment of NK cytotoxicity in patients with B-CLL Chronic exposure to B-CLL tumour cells within the circulation and high levels of TGF-β1 may serve to impair NK cell function in patients with B-CLL.

One of our most striking and novel observations was the profound difference in the profile of NK cell populations taken from patients with B-CLL or SLL. Indeed, NK cells from patients with SLL showed no significant impairment in function or phenotype compared to cells taken from HD. The clinical difference between B-CLL and SLL is the tissue distribution of the tumor cells. SLL is characterized by lymphadenopathy and/or splenomegaly in the absence of a peripheral lymphocytosis, whereas B-CLL is defined by the presence of tumor cells in the blood. As such, NK cells in patients with B-CLL will be exposed continually to tumor cells within the circulatory system where chronic signaling interactions, such as tumor cell expressing and shedding NKG2D and NCR ligands, will lead to NKG2D downregulation and potential NK ‘exhaustion’ [14, 34]. Of note we did not find any difference in the level of soluble NKG2D ligands within serum between HD and patients with B-CLL (Supplementary Figure S9). Interestingly, a murine model of NK cell adoptive transfer has also demonstrated the rapid down-regulation of activating receptors in a pattern that correlated with tumor exposure [35]. An overview of potential mechanisms is shown in Figure 6.

Our observations further suggest that NK cells do not interact significantly with tumor cells within lymph nodes in patients with SLL. During health, the great majority of NK cells circulate within the blood and demonstrate a cytolytic CD56^dim^ phenotype. However a population of CD56^hi^ NK cells is observed within lymph nodes where they differentiate from a CD34^dim^CD45RA^+^ hematopoietic precursor cell under the influence of IL-12 or IL-15 [36–38]. We performed immunohistochemical analysis to determine NK cell expression in lymph node sections taken from patients with SLL (*n* = 6) or B-CLL at stage B disease (*n* = 2). Interestingly, CD56^+^ or CD16^+^ NK cells were completely absent in tumor lymph nodes, a pattern that was in marked contrast to the profile observed in healthy lymph node sections and has not previously been reported. No differences were observed between lymph node sections of SLL and B-CLL.

GO (Gene ontology terms) analysis of the microarray result from DAVID functional annotation tool showed that there were 32 genes involved in cell migration, cell motion and chemokine signalling pathway are significantly modulated in B-CLL patients group compared with HD (Supplementary Figure S10). 7 genes, *VAV1, PRKCA, EPHA4, SELPLG, ID1, MYH9* and *CXCR3*, were consistently modulated in both the B-CLL and SLL groups. Several of these proteins are involved in mediating signaling pathways that activate the actin cytoskeleton and cell migration. Two particularly interesting genes are *CXCR3* and *SELPLG* (CD162) which play an important role in the migration of NK cells between the lymphoid compartment and peripheral tissues [39]. Together our data indicate that NK cells are largely unable to enter lymph nodes containing B-CLL/SLL tumor cells and that this helps to preserve peripheral NK cell function in patients with SLL.

These data suggest that the function of the immune system is significantly less impaired in patients with SLL compared to those with B-CLL. Interestingly there has been relatively little study of comparative immune function and relative infection risk in patients with these two disorders. Reduced levels of IgM and IgG are observed in patients with SLL although the IgA concentration is well preserved [40], a factor that has been recently correlated with reduced infection risk in patients with B-CLL who have been treated with ibrutinib [41]. In contrast, the frequency of auto-immune hemolysis and thrombocytopenia is markedly less common in SLL but previous work has not found any evidence of difference in the T cell compartments between the two disease subsets [8, 42]. Although there are no epidemiological studies of the incidence of infectious complications, the widespread clinical impression is that these are less common in patients with SLL, and indeed other patients with low grade non Hodgkins lymphoma, than in patients with B-CLL. To our knowledge this work represents the first comparison of NK cell function from patients with SLL and B-CLL and suggests that preservation of NK cell function could play an important role in maintaining immune homeostasis in this disorder.

Strikingly, the changes described in this work were identified in patients with early stage B-CLL, in whom a ‘watch and wait’ approach to treatment is almost universally adopted. The findings add to the knowledge that clinical observation of early stage B-CLL is associated with gradual impairment of immune function [43] and may contribute to arguments in favour of the early control of the tumor clone. Indeed, downregulation of NKG2D on NK cells has been correlated with disease progression [16] but no such association was observed in our cohort due to the relatively early clinical stage of the patient group. The data are also of interest in relation to the use of signal transduction inhibitors, such as ibrutinib or idelalisib, which can induce a state of lymphocytosis for several months after treatment and which might therefore lead to further impairment of NK function. This suggests that combination therapy with agents such as anti-CD20 antibodies, which control drug-induced lymphocytosis, may help to support NK cell function. However, further studies are needed in this regard and information to date has suggested that infectious complications and immune dysfunction are actually reduced in patients who are treated with these agents [41]. Our data suggests that interventions to improve NK cell function could be a promising strategy to improve the clinical outcome for patients with B-CLL. Indeed, it has been suggested that some of the clinical efficacy of lenalidomide in B-CLL derives from its ability to increase the expression of NKp30 on NK cells and increase tumor cell lysis [44]. In addition recombinant IL15 has also been shown to be presented by B-CLL tumor cells to NK cells *in vitro* where it leads to NK activation and subsequent tumor cell lysis [45].

In summary, we have demonstrated a profound functional impairment of cytotoxic activity in NK cells from patients with B-CLL and have shown for the first time using transcription profiling, that this arises as a result of a global impairment of several biochemical pathways within the cytotoxic machinery. Additionally, we show this pattern is not present in patients with SLL, indicating that NK cell function is determined primarily by the anatomical site of the tumor clone. This observation has significant implications for the development of future clinical protocols, which seek to optimize immune function in the management of B-CLL.

## MATERIALS AND METHODS

### Participants

Patients with B-CLL were recruited from clinics at Birmingham Heartlands Hospital and University Hospital Birmingham over a 6 month period. Patients were assessed by an experienced Hematologist and fulfilled IWCLL criteria for B-CLL or WHO criteria for SLL. 23 Patients with B-CLL were confirmed to be untreated and Binet stage A at recruitment, with a median age of 70 years (IQR 63–81). 8 untreated Patients with SLL were also studied, with a median age of 70 years (IQR 59.8–76.3) and either palpable or CT evidence of lymphadenopathy which was histologically proven to be SLL, in the absence of a peripheral lymphocytosis. All patients provided written informed consent in accordance with ethical approval obtained from the local regional ethics committee (REC no 10/H1206/58). For comparative analysis, 23 healthy age-matched donors were recruited as part of the ongoing 1000 elders recruitment at University of Birmingham, which recruits local healthy individuals who are over the age of 65. Their median age was 73 years (IQR 67–83.2). Written informed consent was obtained and 50 ml of peripheral blood donated (REC no 2002/073).

### Immunophenotyping

Following all blood donations, peripheral blood mononuclear cells (PBMCs) were extracted over a ficoll density gradient and stored at −160°C. PBMCs were later defrosted and used for functional NK cell studies, immunophenotyping and NK cell enrichment prior to microarray analysis.

After defrosting, PBMCs were washed and re- suspended at 10^6^cells/100 μl before staining with live/dead^®^ red stain dye (Invitrogen; Massachusetts, USA). Cells were subsequently washed before incubation with one of the following antibody panels: NKG2D panel; anti-γδ PE, anti-CD3 FITC (Beckman Coulter, USA); anti-CD8 Amcyan, anti-NKG2D APC (BD bioscience, USA), anti-CD4 Percp-Cy5.5 (eBioscience, USA) anti-CD56 PE-Cy7 (Biolegend, USA). DNAM panel; anti-γδ PE; anti-CD8 Amcyan, anti-CD4 Percp-Cy5.5, anti-CD56 PE-Cy7, anti-DNAM FITC, (Biolegend) anti-CD3 APC-Cy7 (BD bioscience) and anti-CD19 APC (eBioscience). NCR panel; anti-γδ PE; anti-CD8 Amcyan, anti-CD4 Percp-Cy5.5, anti-CD56 PE-Cy7, anti-CD3 APC-Cy7, anti-NKp46 Pacific Blue and anti-NKp30 APC (Biolegend,). All flow data unless specified was collected using the BD LSR II flow cytometer and analysed using BD FACSDiva^®^ software (BD Biosciences) (gating strategy in Supplementary Figure S2). For comparison of phenotyping expression data between SLL, B-CLL and HD, non-parametric Mann-Whitney testing was performed. All analysis was performed using Prism version 6.0, Graphpad software, San Diego, USA.

### *In vitro* NK cell cytotoxicity

NK cells were purified using EasySep™ Human NK cell enrichment kit (Stem Cell Technologies, Canada) (purity of sorted NK was around 90–95% of CD3 negative and CD56 positive lymphocytes) and activated overnight at 37°C with Interferon-alpha (IFNα) (PeproTech, USA). For the K562 cytotoxicity assay, K562 cells were labeled with CFSE dye. The labeled K562 cells were then either incubated with RPMI (negative control) or in combination with activated NK cells at an E/T ratio of 0.5:1 on a 96 well plate for 16 hours. Cells were subsequently extracted and a fixed volume analyzed on the BD Accuri™ flow cytometer (BD Bioscience) to gain a relative cell count. % specific lysis was calculated by 100 × {1 – [(experimental group cell count)/(control cell count)]}. Propidium iodide dye was used for gating of the live and dead populations.

For the NKG2D specific NK cytotoxicity assay, a 50:50 mixture of NKG2D ligand (ULBP6 (GenBank ID: AY039682.1) transfected Chinese hamster ovary (CHO) cell line labeled with carboxyfluorescein diacetate succinimidyl ester (CFSE) and control transfected CHO cells labeled with 670 dye, were co-incubated with effector cells (IFN-α activated PBMCs) for 16hrs. After co-culture, the ratio of CFSE negative (670 dye positive, control CHO cells) to CFSE positive cells (ULBP6 transfected CHO cells) was calculated to determine the percentage of specific killing. % specific lysis was calculated by 100 × {1 − [(control ratio)/(experimental ratio)]}. The control ratios refer to the CFSE negative/CFSE positive without effectors cells, while the experimental ratio refers to the CFSE negative/CFSE positive with effectors cells. NKG2D blocking antibody (MAB139) was purchased from R&D system, and used at the concentration of 10 ug/ml in the blocking experiment.

For the rituximab-induced NK cytotoxicity assay, target cells including primary B cells from HD and the B-CLL primary tumor cells were enriched using EasySep™ Human B cell enrichment kit (Stem Cell Technologies, Canada). The target cells were then either incubated with RPMI (negative control) or in combination with activated NK cells with or without Rituximab, at an E:T ratio of 5:1 on a 96 well plate for 16 hours. Cells were subsequently extracted and stained with anti-CD5-PE (Biolegend) and anti-CD19-FITC (Biolegend) to gate the B-CLL primary tumor cells and analysed on the BD Accuri™ flow cytometer to gain a relative cell count. The percentage of specific lysis was then calculated. Propidium iodide dye was used for gating of the live and dead populations.

### *In-vivo* NK cytotoxicity assay using xenograft NOD/SCID mouse model

The animal work was carried out under the Project license: 70/7793 and Personal License number: I031C5AD2. NOG (NOD/Shi-scid/IL-2Rg) mice were bred and maintained at the animal facility, University of Birmingham. The mice were treated for one week with baytril before tumor injection. At 8 weeks old, 12 NOG mice were irradiated with 1.25Gy. They were then injected subcutaneously with 1 × 10^7^ K562 cells to the dorsal lateral thorax.

At day 3, the mice were divided into 3 groups. Each group consisted of 4 mice and received either IL-2 only, IL-2 with 2 × 10^6^ NK cells from HD or IL-2 with 2 × 10^6^ NK cells from Patients with B-CLL. The NK cells were injected intravenously through the tail vein.

The mice were culled seventeen days after receiving the K562 cells. The subcutaneous tumors were then removed and fixed in formaldehyde. After fixation, tissues were cut into sections of 5 μm with a cryomicrotome and then paraffin embedded (performed by Queen Elizabeth Hospital UK). The slides were then deparaffinised and rehydrated. Antigen retrieval was performed using a citrate and EDTA buffer. Slides were then washed with a blocking agent and incubated with primary antibodies. Four primary antibodies were used: anti-CD16, anti-CD56, anti-CD117 and anti-glycophorin C (Abcam, Cambridge, UK). After incubation, the slides were washed with PBS and monoclonal rabbit secondary antibodies were applied (Vector Laboratories, Burlingame CA, USA). The DAB solution (substrate buffer, DAB and chromogen) was then added before submerging in haematoxylin to counterstain nuclei. Images were obtained using a Nikon Eclipse 800 microscope. Tonsil controls were also used to optimize primary antibody concentrations and as a positive control.

### NK cell sorting and microarray gene transcription profiling

NK cells were first isolated from PBMCs using EasySep™ Human NK cell enrichment kit (STEMCELL biotechnology). The magnetically isolated NK cells were stained with anti-CD3 FITC, anti-CD56 Pe-Cy7 and LIVE/DEAD^®^ red stain for a further enrichment, whereby the CD3 negative and CD56 positive live cells were sorted with MoFlo™ cell sorter (Beckman Coulter). The purity of all the sorted NK population was around 99% of CD3 negative and CD56 positive lymphocytes. The sorted NK cell populations were sent to AROS Applied Biotechnology A/S (Aarhus N, Denmark) as dry cell pellets. Total RNA was extracted, labelled and hybridized to GeneChip^®^ Human Transcriptome Array 2.0 (Affymetrix, USA). Microarray data are available in the ArrayExpress database (www.ebi.ac.uk/arrayexpress) under accession number E-MTAB-4403. Raw data was processed using Affymetrix's Expression Console software using default RMA parameters. Statistical analysis of expression data and heatmap generation was performed using d-CHIP software (http://www.dchip.org). The T test in d-CHIP is Welch modified two-sample *t-test*. Multiple comparison adjustment was carried out to include permutation to make sure the median FDR (False Discovery Rate) was < 5%. Venn diagrams were constructed from up or downregulated gene expression in B-CLL or Patients with SLL compared to HD.

## SUPPLEMENTARY MATERIALS FIGURES


