# Supplementary material for: NK cell function is markedly impaired in patients with chronic lymphocytic leukaemia but is preserved in patients with small lymphocytic lymphoma
Source: Oncotarget. 2016 Sep 17;7(42):68513–26. doi: 10.18632/oncotarget.12097 (PMC5356570; doi:10.18632/oncotarget.12097)
Supplement: Supplementary file 1 [file oncotarget-07-68513-s001.pdf]

NK cell function is markedly impaired in patients with chronic lymphocytic leukaemia but is preserved in patients with small lymphocytic lymphoma

Supplementary Materials

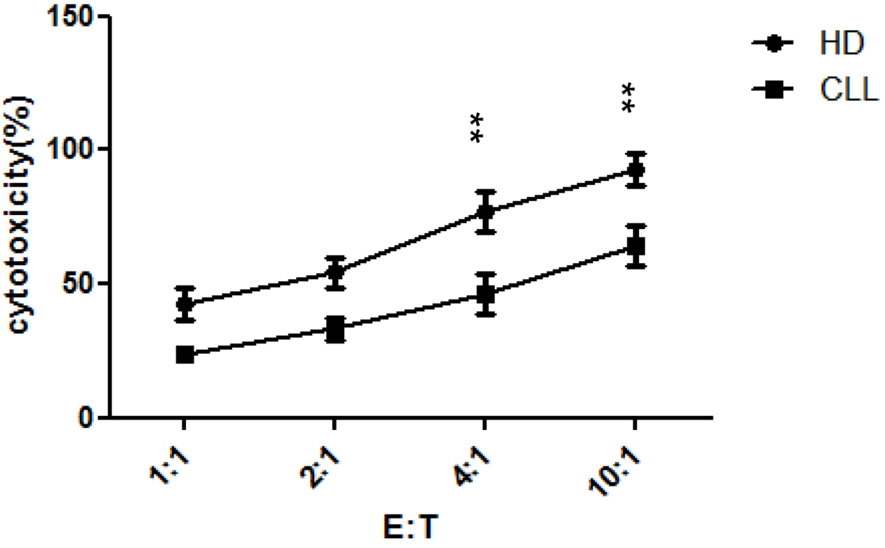

Supplementary Figure S1: The cytotoxicity to K562 cells is reduced by NK cells from patients with B-CLL. NK cells were enriched and the cytotoxicity to K562 cells were measured using DELFIA® EuTDA Cytotoxicity Reagents (AD0116 PerkinElmer Inc). Briefly, K562 cells were first loaded with an acetoxymethyl ester of BATDA before they were co-cultured with enriched NK cells from HD and B-CLL patients at a different effector/target(E/T) ratio for 4 hrs. TDA release after target cell lysis was detected by addition of Europium solution and measurement of the fluorescence signal. Specific cytotoxicity was calculated using the formula: % Specific release = (Experimental release (counts) − Spontaneous release (counts))/(Maximum release (counts) − Spontaneous release (counts))× 100. Data are shown as mean values of the specific cytotoxicity from 3 HD and 3 patients with B-CLL. Error bars represent standard errors and significance was determined using two-way ANOVA, $p < 0.01$(**).

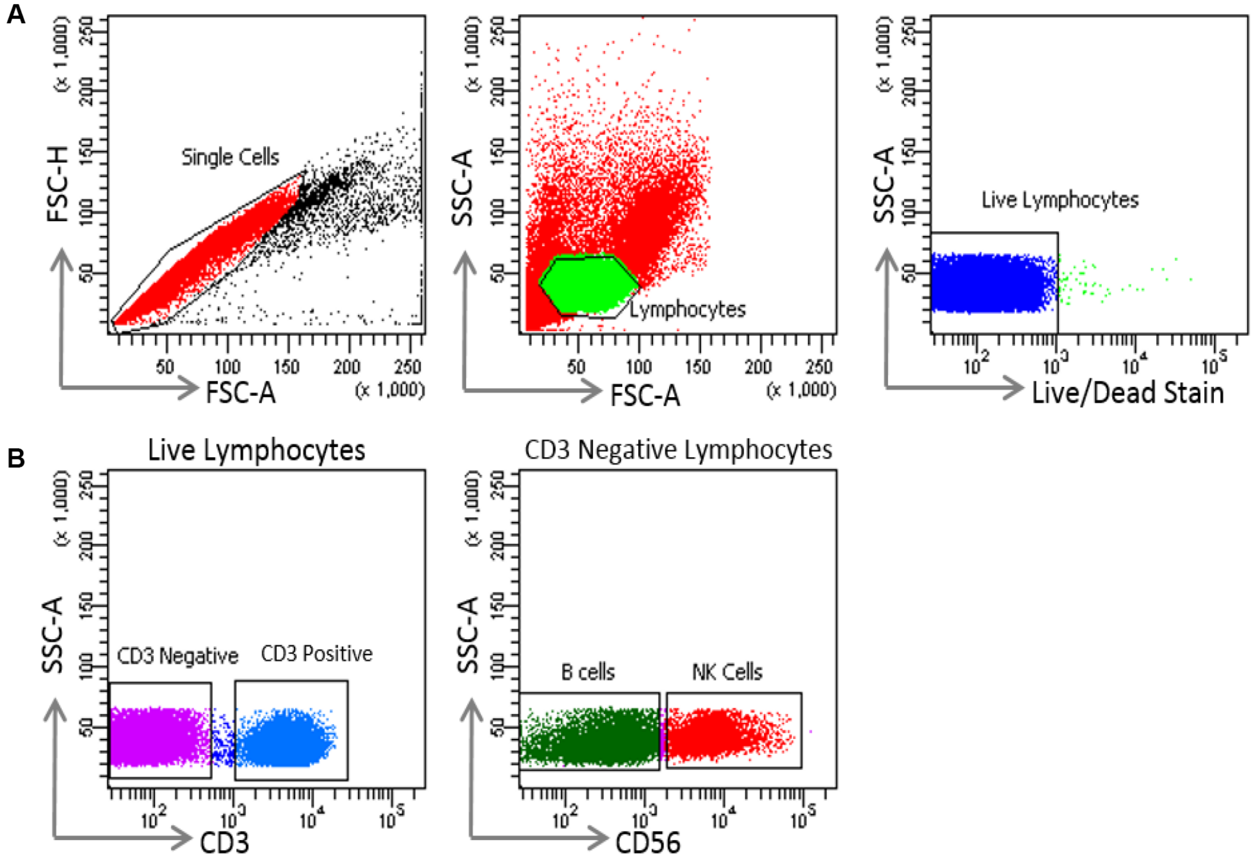

Supplementary Figure S2: Flow cytometry gating strategy. Single cells were firstly identified from whole PBMCs. Gated live lymphocytes were then used to identify NK cell populations as CD3 negative, CD56 positive cells.

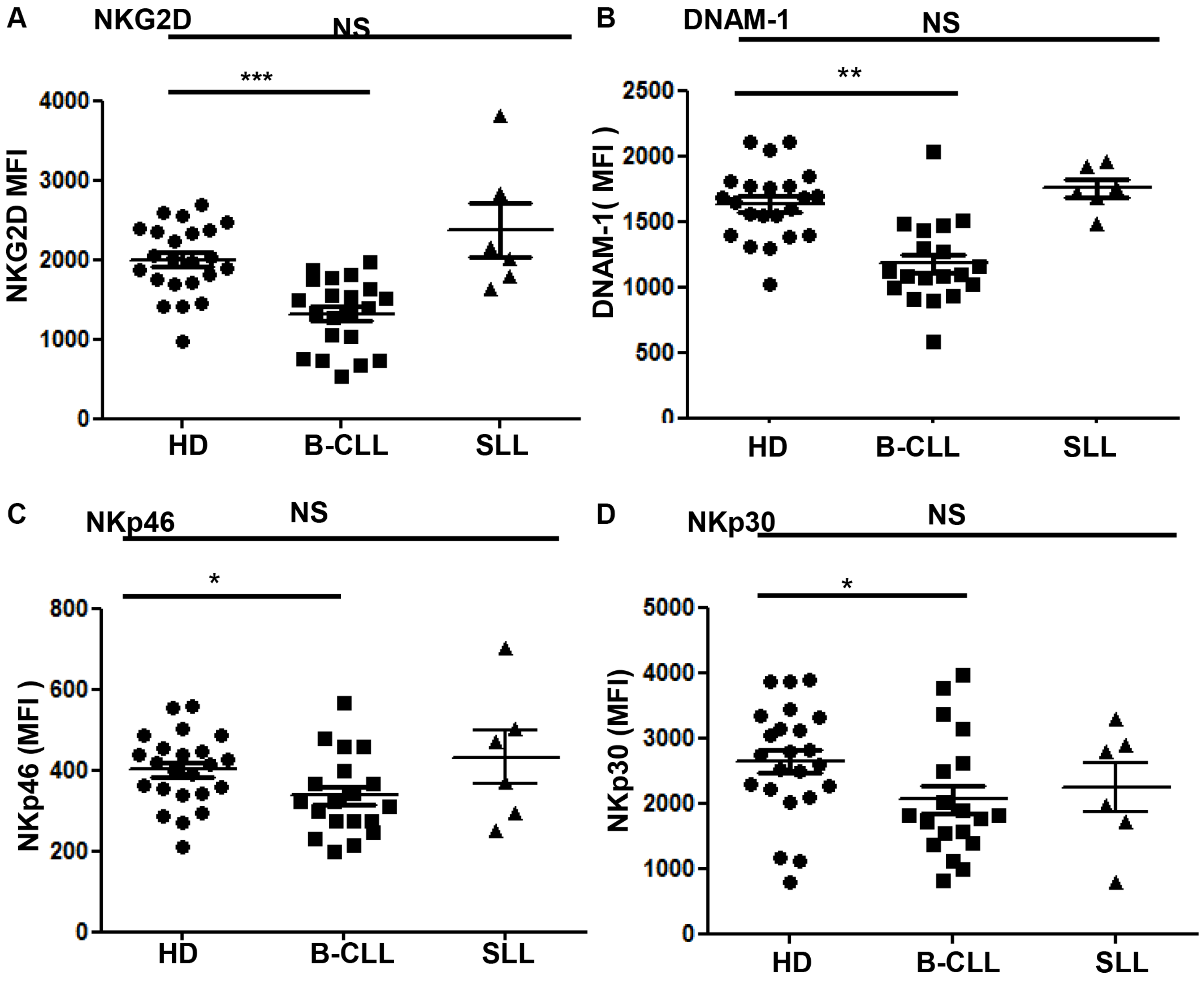

Supplementary Figure S3: NKG2D, DNAM-1, NKp46 and NKp30 receptors on NK cells are downregulated in patients with B-CLL but not patients with SLL. The NKG2D (**A**), DNAM-1 (**B**), NKp46 (**C**) and NKp30 (**D**) surface expression on NK populations from HD, patients with B-CLL and patients with SLL were studied using flow cytometry. Data shown are mean values for mean fluorescence intensity(MFI) of surface staining. Error bars represent standard errors and significance was determined using Mann-Whitney testing, $p < 0.05$(*) , $p < 0.01$(**) and $p < 0.001$(***).

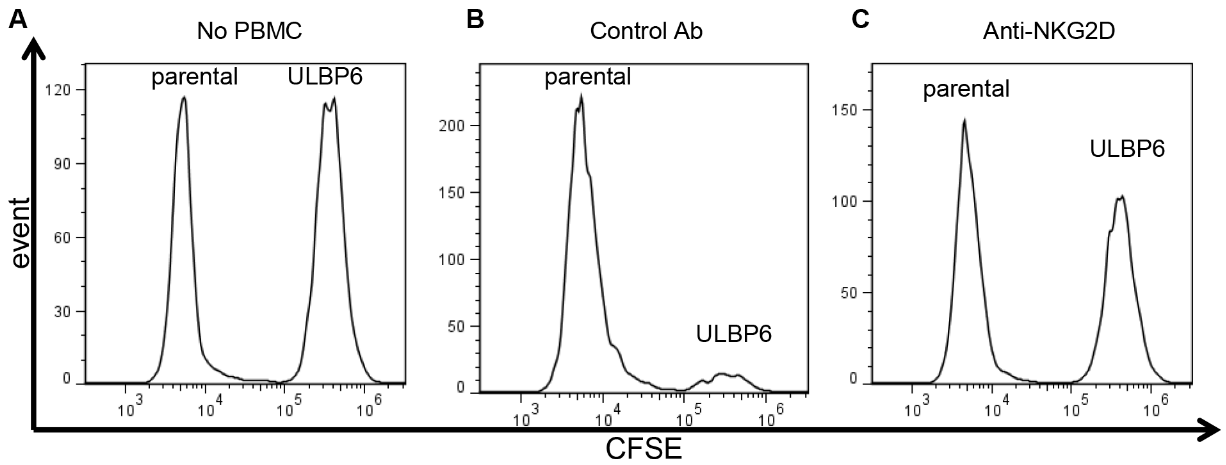

Supplementary Figure S4: The cytotoxicity to CHO-ULBP6 is NKG2D dependent. PBMC-based assay of specific cytotoxic responses to CHO cells expressing ULBP6 in a representative healthy donor, in the presence (**C**) or absence (**B**) of blocking anti-NKG2D antibody.

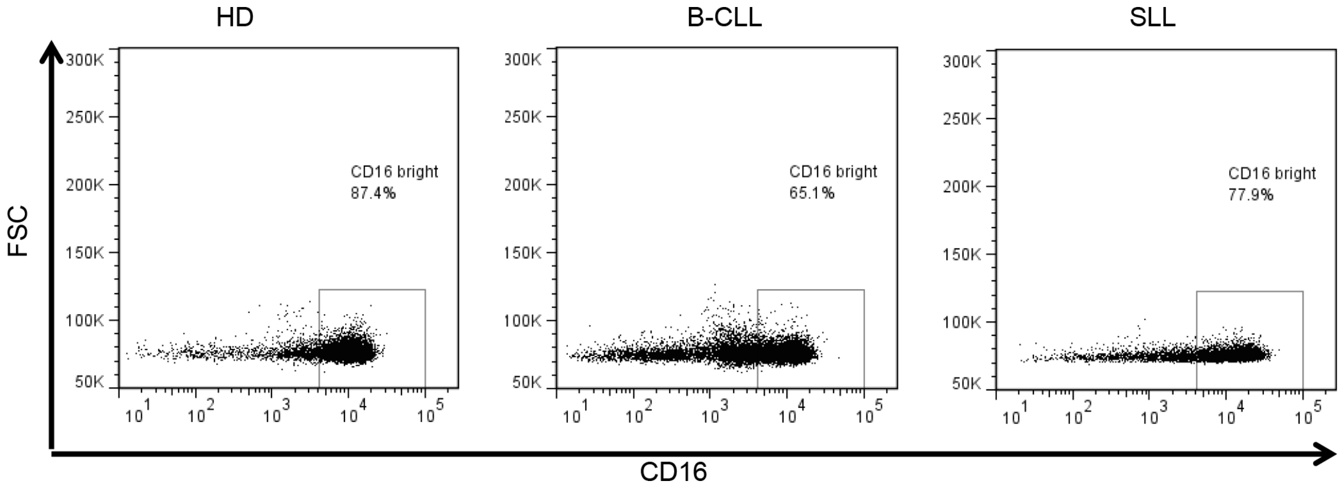

Supplementary Figure S5: Gating strategy for CD16^{bright} . NK cells identified from PBMCs using CD56 and CD3. Then CD16 bright population was gated according to CD16 staining pattern on the NK cell populations.

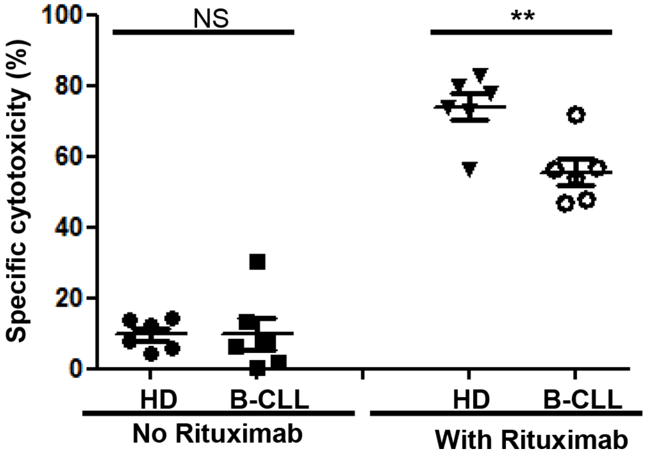

Supplementary Figure S6: Rituximab induced NK cytotoxicity assay using B cells from healthy donor as target. B cells from HD were enriched and incubated with NK cells from HD or patients with B-CLL, with or without Rituximab, for 16 hours. The specific lysis was calculated according to the relative cell counting using flow cytometry. Data shown are mean values of the specific cytotoxicity by NK cells, error bars represent standard errors and significance was determined using Mann-Whitney testing, $P < 0.01$(**).

Phosphprotein(38)

Gene symbol	description	CLL(Fold changes)	SLL(Fold changes
ADAM9	metallopeptidase domain 9	-1.624341	-1.515804
CD69	CD69 molecule	-1.556424	-1.670129
ARHGAP31	Cdc42 GTPase-activating protein	-4.258728	-5.377674
FOSL2	FOS-like antigen 2	-1.640036	-1.59406
GRASP	GRP1 (general receptor for phosphoinositides 1)-associated scaffold protein	-2.272311	-1.874172
NECAP1	NECAP endocytosis associated 1	1.554797	1.67798
SAR1A	SAR1 homolog A	1.517245	1.619355
SKIL	SKI-like oncogene	-2.27814	-1.785319
WWC2	WW and C2 domain containing 2	-1.949762	-1.703699
ANKRD28	ankyrin repeat domain 28	2.301504	1.614136
AUTS2	autism susceptibility candidate 2	-2.210972	-2.062404
BAZ1A	bromodomain adjacent to zinc finger domain, 1A	-1.821478	-2.050731
CREB1	cAMP responsive element binding protein 1	-1.839904	-1.770372
CREM	cAMP responsive element modulator	-1.665931	-1.858836
CASP8	caspase 8, apoptosis-related cysteine peptidase	1.525664	1.622755
C1orf21	chromosome 1 open reading frame 21	-1.801542	-1.661947
C16orf54	chromosome 16 open reading frame 54	2.001255	1.589108
C5orf25	chromosome 5 open reading frame 25	-1.652645	-1.661897
DUSP16	dual specificity phosphatase 16	-1.827226	-1.699478
FAM177A1	family with sequence similarity 177, member A1	-1.951131	-1.912894
GLG1	golgi apparatus protein 1	-1.943558	-1.555048
HSF2	heat shock transcription factor 2	1.661108	1.811061
HIST1H3A	histone cluster 1	-1.931873	1.70527
HIST1H3B	histone cluster 1	-1.693491	2.056228
IGF1R	insulin-like growth factor 1 receptor	-1.685208	-1.68879
ITGA6	integrin, alpha 6	-3.467409	-1.786681
IRF2BP2	interferon regulatory factor 2 binding protein 2	-1.605661	-1.750973
MED14	mediator complex subunit 14	1-.569984	-1.511179
MYADM	myeloid-associated differentiation marker	2.117809	1.849359
NR1D1	nuclear receptor subfamily 1, group D, member 1	1.617725	1.775971
PLN	phospholamban	1.812395	2.523499
PHLDB2	pleckstrin homology-like domain, family B, member 2;	-3.350928	-2.411696
KCTD10	potassium channel tetramerisation domain containing 10	-1.814611	-1.826904
SUDS3	suppressor of defective silencing 3 homolog	1.703513	1.630459
TXNIP	thioredoxin interacting protein	1.613654	2.011565
VAV1	vav 1 guanine nucleotide exchange factor	-1.69213	-1.917351
ZFP36	zinc finger protein 36	-1.717131	1.558329
ZMYND11	zinc finger, MYND domain containing 11	-1.56747	-1.578275

Intracellular signalling cascade(11)

Gene symbol	description	CLL(Fold changes)	SLL(Fold changes
ADAM9	metallopeptidase domain 9	-1.624341	-1.515804
ARHGAP31	Cdc42 GTPase-activating protein	-4.258728	-5.377674
SAR1A	SAR1 homolog A	1.517245	1.619355
CREM	cAMP responsive element modulator	-1.665931	-1.858836
DUSP16	dual specificity phosphatase 16	-1.827226	-1.699478
GADD45B	growth arrest and DNA-damage-inducible, beta	-2.248157	-1.937782
IGF1R	insulin-like growth factor 1 receptor	-1.685208	-1.68879
MED14	mediator complex subunit 14	-1.569984	-1.511179
PHLDB2	pleckstrin homology-like domain, family B, member 2	-3.350928	-2.411696
VAV1	vav 1 guanine nucleotide exchange factor	-1.69213	-1.917351
ZFP36	zinc finger protein 36, C3H type	-1.717131	1.558329

Apoptosis(9)

Gene symbol	description	CLL(Fold changes)	SLL(Fold changes
ADAM9	metallopeptidase domain 9	-1.624341	-1.515804
NLRP3	NLR family, pyrin domain containing 3	-1.844568	-1.658023
CREB1	cAMP responsive element binding protein 1	-1.839904	-1.770372
CASP8	caspase 8, apoptosis-related cysteine peptidase	1.525664	1.622755
CUL4A	cullin 4A	-2.061744	-1.691255
IGF1R	insulin-like growth factor 1 receptor	-1.685208	-1.68879
SERPINB9	serpin peptidase inhibitor, clade B	1.738767	1.936593
TXNIP	thioredoxin interacting protein	1.613654	2.011565
VAV1	vav 1 guanine nucleotide exchange factor	-1.69213	-1.917351

Transcription regulation(15)

Gene symbol	description	CLL(Fold changes)	SLL(Fold changes
SKIL	SKI-like oncogene	-2.27814	-1.785319
AHR	aryl hydrocarbon receptor	2.199513	2.401935
BRMS1L	breast cancer metastasis-suppressor 1-like	-1.580083	1.658639
BAZ1A	bromodomain adjacent to zinc finger domain, 1A	-1.821478	-2.050731
CREB1	cAMP responsive element binding protein 1	-1.839904	-1.770372
CREM	cAMP responsive element modulator	-1.665931	-1.858836
HSF2	heat shock transcription factor 2	1.661108	1.811061
IRF2BP2	interferon regulatory factor 2 binding protein 2	-1.605661	-1.750973
JMJD6	jumonji domain containing 6	-2.100287	-1.62571
MED14	mediator complex subunit 14	-1.569984	-1.511179
NR1D1	nuclear receptor subfamily 1, group D, member 1	1.617725	1.775971
SUDS3	suppressor of defective silencing 3 homolog	1.703513	1.630459
TXNIP	thioredoxin interacting protein	1.613654	2.011565
ZNF710	zinc finger protein 710	-1.652275	-2.033264
ZMYND11	zinc finger, MYND domain containing 11	-1.56747	-1.578275

others(47)

Gene symbol	description	CLL(Fold changes)	SLL(Fold changes
ALOX5AP	Arachidonate 5-Lipoxygenase-Activating Protein	-1.735243	-1.831961
ANKH	Inorganic Pyrophosphate Transport Regulator	-2.077653	-1.712332
ARSD-AS1	ARSD Antisense RNA 1	-2.785523	-1.63
C10orf31	CELF2 antisense RNA 1	2.423804	1.928385
DAAM1	Dishevelled Associated Activator Of Morphogenesis	-1.75864	-1.645267
DNAJB7	DnaJ (Hsp40) Homolog, Subfamily B, Member 7	1.682713	1.575715
DTHD1	Death Domain Containing 1	1.710254	1.663715
FAM105A	Family With Sequence Similarity 105, Member A	-1.853797	-1.564916
FAM105B	Family with sequence similarity 105, member B	-2.468972	-2.330079
GPR174	Probable G-protein coupled receptor 174	2.164354	1.810403
HSD17B12	Estradiol 17-beta-dehydrogenase 12	-1.690102	-1.513342
IER2	immediate early response 2	-1.56747	-1.684103
IGHD2-21	immunoglobulin heavy diversity 2-21	-1.79005	2.531513
JAG1	jagged 1 protein	-1.750184	-2.061796
LOC100131096	TNRC6C antisense RNA 1	1.60214	1.547565
LOC100506647	uncharacterized LOC100506647	2.056228	1.536875
LOC202181	SUMO-interacting motifs containing 1 pseudogene	-1.566997	-1.675314
METRNL	meteorin, glial cell differentiation regulator-like	-1.564501	-1.652475
MGC57346	ADP-ribosylation factor pseudogene	1.660398	2.175902
MIR1200	microRNA 1200	-3.606001	-1.582421
MIR4441	microRNA 4441	-2.639016	2.479415
MIR643	microRNA 643	1.881437	2.653125
MRPL10	mitochondrial ribosomal protein L10	1.50759	1.700504
PLIN2	perilipin 2	2.049041	1.630855
PPP1R14BP3	protein phosphatase 1, regulatory (inhibitor) subunit 14B pseudogene 3	-1.662206	-1.798865
RN5S196	RNA, 5S ribosomal pseudogene 196	-1.670176	1.866066
RN5S212	RNA, 5S ribosomal pseudogene 212	1.984273	2.98566
RN5S229	RNA, 5S ribosomal pseudogene 229	1.632762	1.700264
RN5S268	RNA, 5S ribosomal pseudogene 268	2.769351	6.03132
RN5S368	RNA, 5S ribosomal pseudogene 368	1.518613	1.788121
RN5S371	RNA, 5S ribosomal pseudogene 3671	-3.451426	-3.753289
RN5S394	RNA, 5S ribosomal pseudogene 394	2.098807	2.010691
RNU5E-1	RNA, U5E small nuclear 1	-1.591073	1.853176
RNU7-45P	RNA, U7 small nuclear 45 pseudogene	1.91246	1.653218
RNY4P7	RNA, Ro-associated Y4 pseudogene 7	1.52938	2.475524
RSF1-IT2	RSF1 intronic transcript 2	1.759194	1.59446
SIK3-IT1	SIK3 intronic transcript 1	1.517751	1.682244
SLC25A30	solute carrier family 25, member 30	1.958755	2.179846
SNORA12	small nucleolar RNA, H/ACA box 12	1.511508	2.532863
SNORD62B // SNORD62A	small nucleolar RNA, C/D box 62A /B	1.526259	1.526259
SOAT1	sterol O-acyltransferase 1	1.584228	1.822816
TCP11L2	t-complex 11, testis-specific-like 2	-1.855092	-2.161073
TMEM14E	transmembrane protein 14E	2.496681	2.715892
UPF3AP1	UPF3A pseudogene 1	-1.530705	-1.503339
VTRNA1-3	vault RNA 1-3	2.281941	4.980698
WWC2-AS1	WWC2 antisense RNA 1	-1.814426	-1.718231
ZBTB20-AS2	ZBTB20 antisense RNA 2	-2.234574	1.635804

Supplementary Figure S7: The genes that have been significantly modified in both patients with B-CLL or SLL. A list of the 93 genes that are upregulated or downregulated on microarray analysis of NK cells taken from patients with B-CLL or SLL patients compared with HD. This list includes 46 genes which are downregulated in both B-CLL and SLL patients, 36 genes which are upregulated in both B-CLL patients and SLL patients and 11 genes that are differentially regulated in B-CLL and SLL patients. They have been classified using DAVID Gene Functional Classification Tool (https://david.ncifcrf.gov/home.jsp). 38 genes have been identified to be phosphoproteins; 11 genes have been identified to be involved in an intracellular signaling cascade; 9 genes have been identified to be related to regulation of apoptosis and 15 genes have been identified to be related to transcription regulation.

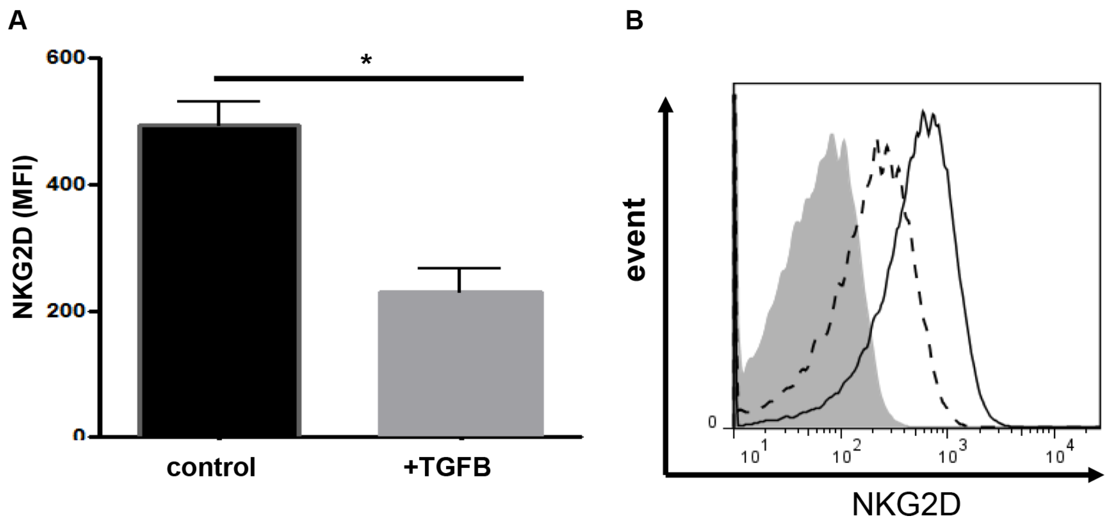

Supplementary Figure S8 :TGFβ can downregulate the NKG2D expression of NK cells. (**A**) NK cells were incubated with TGFβ-1 for 48hrs and surface expression of NKG2D was assessed using flow cytometry. Data shown are mean values of MFI of NKG2D staining from 5 separate donors , error bars represent standard errors and significance was determined using Mann-Whitney tests, $P < 0.01$(**). (**B**) An example histogram plot of NKG2D expression, solid black line represents no TGFβ-1 treatment, dashed black line represents with TGFβ-1 treatment and grey-filled histograms represent isotype control staining.

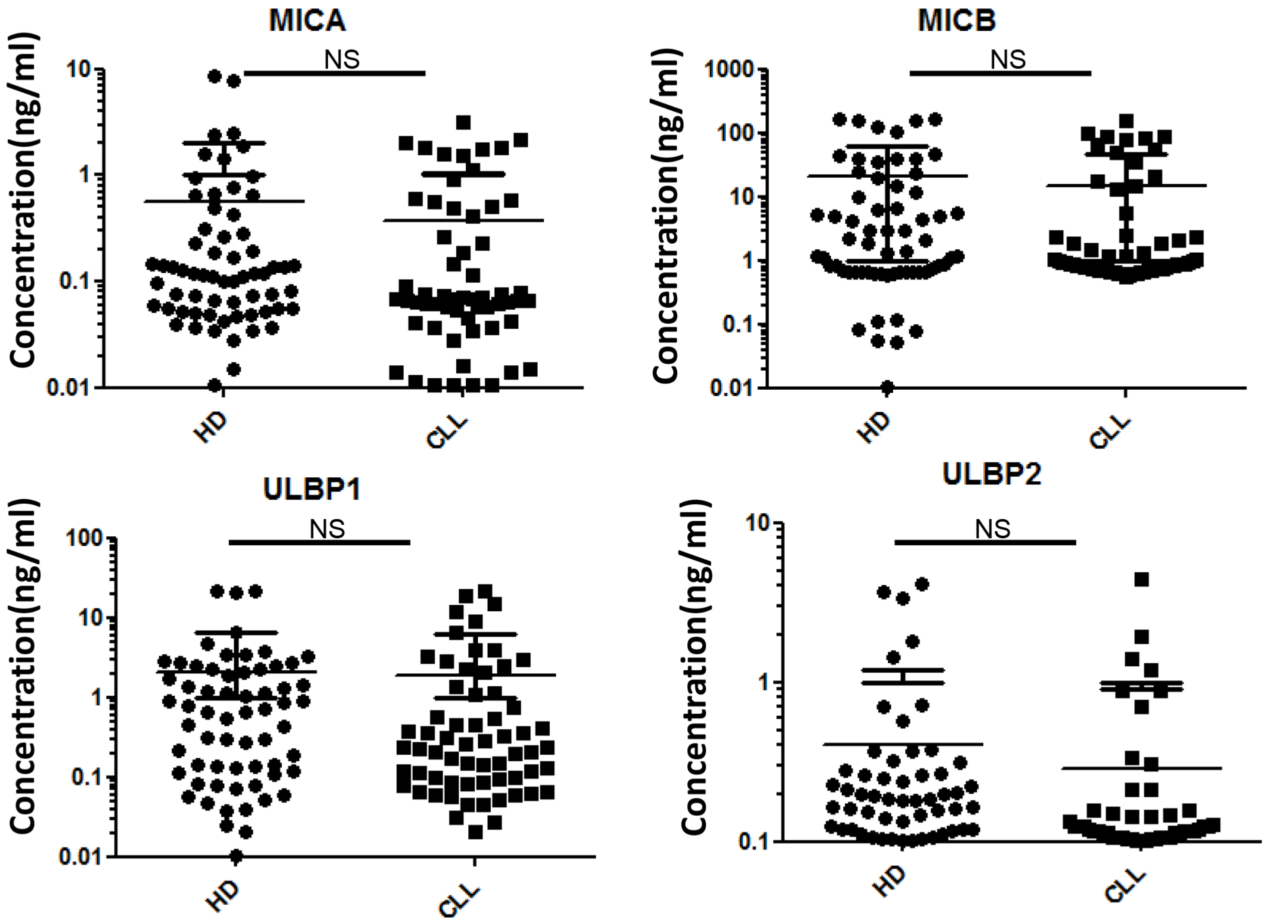

Supplementary Figure S9: The concentration of soluble NKG2D ligands in the serum of patients with B-CLL are not upregulated compared with HD. The serum from 64 healthy donors and 64 patients with B-CLL were tested for concentration of four ligands of NKG2D: MICA, MICB, ULBP1 and ULBP2. ELISA kits were obtained from R&D systems: DY1300, DY1599, DY1380 and DY1298.

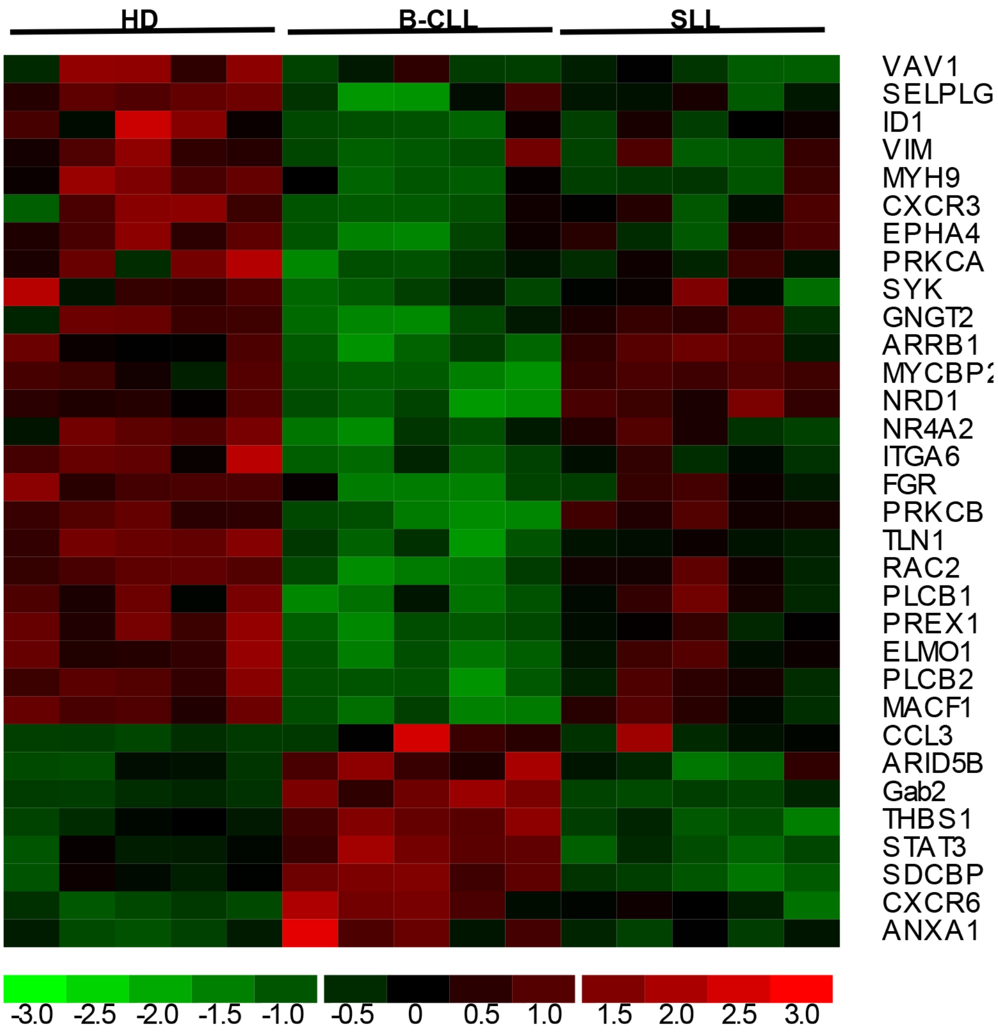

Supplementary Figure S10: The relative intensity profiles of the migration related genes. The relative intensity profiles of 32 genes involved in cell migration, cell motion and chemokine signalling pathway that were significantly modulated in B-CLL patients group compared with HD. Each column represents one sample, while each row represents relative hybridization intensities of an individual gene. The colour on the heatmap indicates the magnitude of the relative expression of genes across the samples, with brighter red indicating higher expression and brighter green indicating lower expression.
